# A55 CHRONIC STRESS IMPAIRS THE INTESTINAL CRYPTAL STEM CELL CYCLE AND PANETH CELL PROLIFERATION

**DOI:** 10.1093/jcag/gwae059.055

**Published:** 2025-02-10

**Authors:** X BAI

**Affiliations:** Medicine, Kyushu Daigaku, Fukuoka, Fukuoka, Japan

## Abstract

**Background:**

The small intestine’s epithelial barrier is essential for intestinal homeostasis and host defense. It consists of epithelial cells constantly renewed by crypt stem cells and their progeny, which have different functions in the gut. Chronic stress can impair the barrier by modulating the gut-brain axis, which involves the hypothalamic-pituitary-adrenal (HPA) axis, the vagus nerve, and the immune system. Chronic stress can also alter the gut microbiota, further influencing the barrier and immune system.

**Aims:**

The present study examines how chronic stress affects the crypt stem cell niche and function in the small intestine.

**Methods:**

Restraint stress was applied to 6-8 weeks-old mice in a plastic container for 1 hour daily for 7 days. The dark/light transition box test evaluated stress-induced behavior change. The Ussing chamber technique was used to assess the paracellular permeability of the small intestine. RNA was extracted from the mucosa and submucosa layers of the small intestine, after removing the muscle layer, and RNA sequencing analysis was performed. Immunofluorescence analysis was also performed on the small intestinal tissues to detect the expression of various markers. To assess the *in vivo* effect of cortisol, dexamethasone (a synthetic steroid activating the same receptors as cortisol), or metyrapone (an 11β-hydroxylation blocker to inhibit cortisol production), was applied. To assess the possible involvement of microbiota, antibiotics were applied before and during the restraint stress.

**Results:**

We found that restraint stress-induced anxiety-like behavior, increased small intestinal epithelial permeability and reduced villus and crypt length in mice. RNA sequencing analysis revealed that restraint stress downregulated the markers of Paneth cells and stem cells in the small intestinal mucosa. Immunofluorescence analysis confirmed that restraint stress reduced the expression of stem cell marker Olfm-4 and proliferation markers Ki-67 and p-H3 in the small intestinal crypts. 16s rRNA analysis showed increased Streptococcus and decreased Bifidobacterium in stressed mice fecal samples. Ablation of gut microbiota by antibiotics attenuated the stress-induced downregulation of stem cell markers but did not affect the Paneth cell markers. Dexamethasone, a synthetic glucocorticoid, mimicked the effects of restraint stress on crypt stem cells and Paneth cells, while metyrapone, a steroid synthesis inhibitor, attenuated both.

**Conclusions:**

Chronic stress induces anxiety, gut microbiota alteration, and barrier dysfunction. The change in epithelial villi and crypts in the small intestine is in a microbiota-dependent manner. These findings show how chronic stress impacts intestinal epithelial renewal and differentiation, which may affect intestinal health and disease.

**Funding Agencies:**

JSPS KAKENHI

